# Impact of Diagnosing Urologists and Hospitals on the Use of Radical Cystectomy

**DOI:** 10.1016/j.euros.2020.06.001

**Published:** 2020-06-23

**Authors:** Vishnukamal Golla, Yong Shan, Hemalkumar B. Mehta, Zachary Klaassen, Douglas S. Tyler, Jacques Baillargeon, Ashish M. Kamat, Stephen J. Freedland, John L. Gore, Karim Chamie, Yong-Fang Kuo, Stephen B. Williams

**Affiliations:** a Department of Urology, University of California, Los Angeles, CA, USA; b Department of Surgery, Division of Urology, The University of Texas Medical Branch, Galveston, TX, USA; c Department of Surgery, Section of Urology, Medical College of Georgia, Georgia Regents University, Augusta, GA, USA; d Department of Surgery, The University of Texas Medical Branch at Galveston, Galveston, TX, USA; e Department of Preventive Medicine and Community Health, Sealy Center of Aging, The University of Texas Medical Branch, Galveston, TX, USA; f Department of Urology, The University of Texas MD Anderson Cancer Center, Houston, TX, USA; g Department of Urology, Cedars Sinai Medical Center, Los Angeles, CA, USA; h Department of Urology, The University of Washington, Seattle, WA, USA

**Keywords:** Bladder cancer, Radical cystectomy, Variation, Utilization

## Abstract

**Background:**

One out of five patients with muscle-invasive bladder cancer undergo radical cystectomy—a guideline-recommended treatment. Previous studies have primarily evaluated patient characteristics associated with the use of radical cystectomy, ignoring potential nesting of data.

**Objective:**

To determine the impact of patient, diagnosing urologist, and hospital characteristics on the variation in the use of radical cystectomy.

**Design, setting, and participants:**

This is a retrospective cohort study using the Surveillance, Epidemiology, and End Results Registry (SEER)-Medicare linked database.

**Outcome measurements and statistical analysis:**

A total of 7097 muscle-invasive bladder cancer patients and 4601 diagnosing urologists affiliated to 822 hospitals from January 1, 2002 to December 31, 2012 were analyzed. Multilevel logistic regression analyses were used to determine variation and factors associated with the use of radical cystectomy.

**Results and limitations:**

Of the 7097 patients, only 27% underwent radical cystectomy. The intraclass correlation coefficient for variation in the use of radical cystectomy attributed to the hospital level was 4.3%. Higher radical cystectomy volume by diagnosing urologists (more than five vs zero to one surgery: odds ratio [OR], 1.27; 95% confidence interval [CI], 1.00–1.62) and hospitals (more than five vs zero to four surgeries: OR,1.48; 95% CI, 1.14–1.93) was associated with increased use of radical cystectomy. Patients diagnosed by female rather than male urologists were more likely to undergo radical cystectomy (OR, 1.32; 95% CI, 1.07–1.62).

**Conclusions:**

We found that 4.3% variation in the use of radical cystectomy was attributed to the hospital level, leaving 95.7% variation in use unexplained. We identified significantly increased use among higher-volume and female diagnosing urologists. These findings support further investigation into measures beyond hospital volume, which largely impact the utilization of radical cystectomy.

**Patient summary:**

In this large population-based study, we found that 4.3% of variation in the use of radical cystectomy was attributed to the hospital level, leaving 95.7% variation in use unexplained. Higher radical cystectomy volume of diagnosing urologists and female urologists were independently associated with increased use of radical cystectomy. These findings support further investigation into measures beyond hospital volume, which largely impact the utilization of radical cystectomy.

## 1 Introduction

There will be approximately 80 470 new cases and 17 670 deaths due to bladder cancer in the USA in 2019 [1]. Radical cystectomy (RC) with extended pelvic lymph node dissection is a guideline-recommended treatment [2]; however, only one out of five patients with muscle-invasive bladder cancer undergo this potentially curative surgery [3], [4].

Patient, physician, and hospital factors can influence the use of radical cystectomy as observed in other high-risk surgical procedures [5]. Prior studies have found that nonmodifiable patient factors such as advanced age, sex, race/ethnicity, and greater comorbidity burden may impact the use of radical cystectomy [3], [4], [6]. Additionally, modifiable risk factors such as higher-volume hospitals, higher-volume urologists, and surgeries performed at academic centers have been associated with increased use of radical cystectomy [3], [6], [7]. While these modifiable risk factors could serve as prime targets for increased utilization of radical cystectomy [8], centralization of care to higher-volume providers remains a debate in the USA, with no change in radical cystectomy use over the last 3 decades [3], [4].

Prior studies have focused mainly on hospital variation in the use of radical cystectomy, with high-volume hospitals demonstrating increased use and decreased morbidity/mortality when surgery was performed at these centers [9]. However, no study to date has assessed the impact of the diagnosing urologist on the use of radical cystectomy. Given the diagnosing urologists’ role as the index provider in the disease pathway, patients are inclined to make decisions regarding treatment based on their urologist's recommendations [10]. Furthermore, the hospital affiliation of these urologists may also impact their recommendation on the use of high-risk procedures. Against this backdrop, we sought to determine variation in the use of radical cystectomy attributed to hospitals, while taking into account the impact of patient and diagnosing urologist characteristics.

## 2 Patients and methods

### 2.1 Data source

We used the Surveillance, Epidemiology, and End Results (SEER)-Medicare linked database. The SEER dataset contained information on a nationally representative sample of patients with newly diagnosed cancers in 18 US regions, which is nearly 35% of the US population. In the USA, Medicare is the federal government program that provides healthcare coverage for those aged 65 yr or older, younger people aged < 65 yr with disabilities, and those with end stage renal disease (permanent kidney failure requiring dialysis or transplant). The study was deemed exempt by the Institutional Review Board at The University of Texas Medical Branch.

### 2.2 Study population

**Fig. 1:**
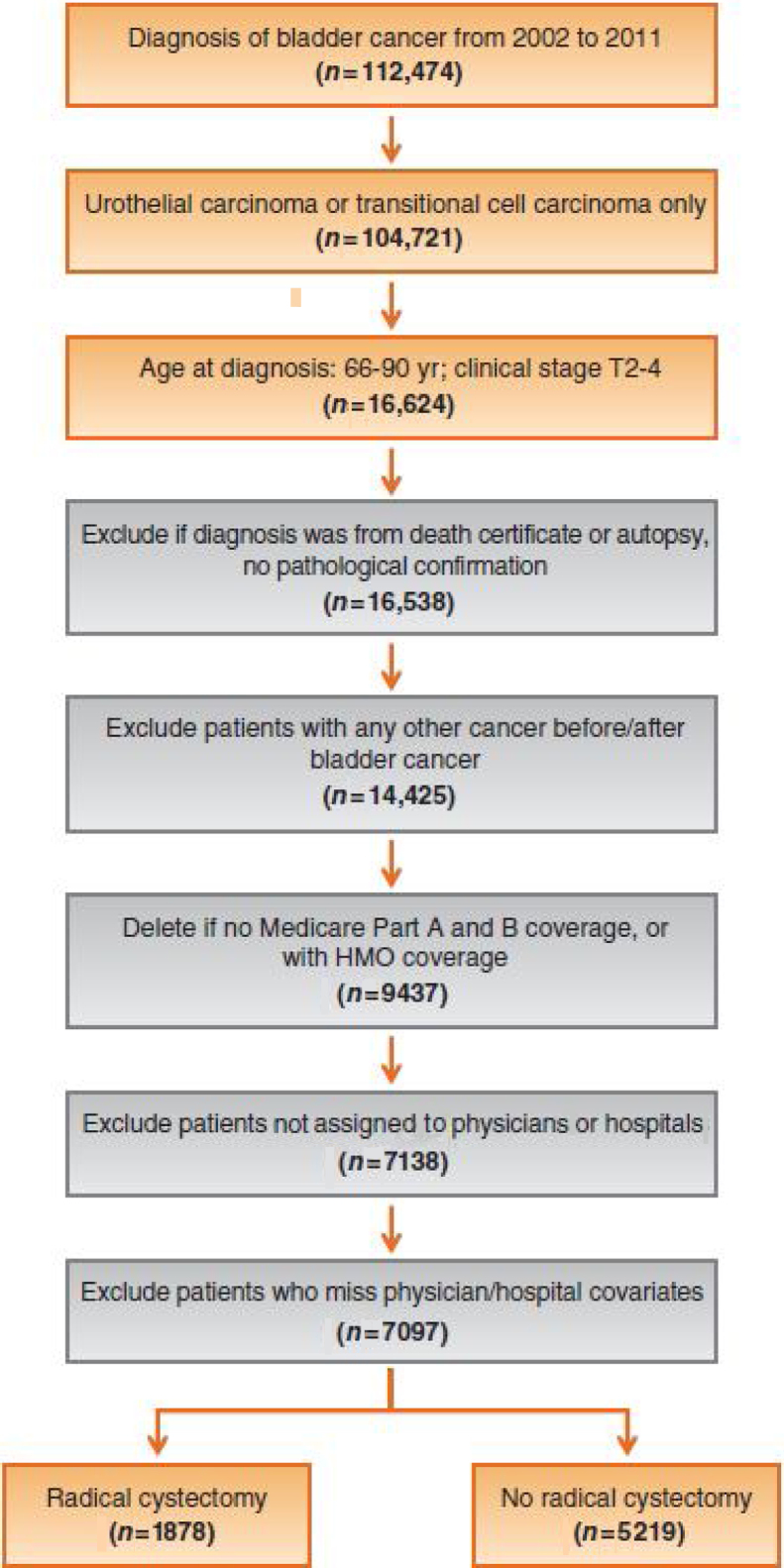
Patient selection process. HMO = Health Maintenance Organization.

The study cohort included patients, 66–85 yr of age, diagnosed with clinical stage T2–T4a, N0, M0 bladder cancer (transitional cell or urothelial carcinoma) from January 1, 2002 to December 31, 2011 (Fig. 1). Medicare claims data were reviewed through December 31, 2012. We included cT2–T4a, N0, M0 patients, as radical cystectomy is a guideline-recommended treatment option for these patients [11]. We excluded patients who did not have a pathologic confirmation of bladder cancer, were diagnosed with bladder cancer postmortem, and had non–bladder cancer malignant diagnoses. We restricted the study sample to patients who had Medicare fee-for-service coverage in the year prior to the diagnosis and continuous enrollment after diagnosis for whom Medicare part A and part B claims data were available. Additionally, we excluded patients who were not assigned to a urologist or hospital.

### 2.3 Identification of radical cystectomy

All patients were followed for 1 yr from the date of cancer diagnosis to identify radical cystectomy use [12]. Patients who underwent surgery alone or in combination with radiation or chemotherapy were included in the radical cystectomy group; all the remaining patients were categorized as those having received no radical cystectomy treatment. Radical cystectomy was identified using the International Classification of Diseases—version 9 (ICD-9) and Common Procedural Terminology codes corresponding to radical cystectomy [3].

### 2.4 Study covariates

#### 2.4.1 Patient characteristics

Information on patient age, sex, race/ethnicity (non-Hispanic white, non-Hispanic black, Hispanic, and non-Hispanic other races), marital status (single, married, and unknown), region (northeast, south, midwest, and west), and socioeconomic characteristics was extracted from the SEER-Medicare linked database. Education was defined according to the percentage of patients with a least 4-yr college education categorized into quartiles. Comorbidity was assessed using the Klabunde modification of Charlson Comorbidity Index (CCI) the year before cancer diagnosis [13].

#### 2.4.2 Characteristics of diagnosing urologist

Diagnosing urologists were defined as the index providers who performed the initial transurethral resection of the bladder tumor and made the first diagnosis of bladder cancer from Medicare claims. When patients were seen by two or more urologists, the patient was assigned to the urologist with 75% or more of the urologist visits in the 1st year before diagnosis [14]. Characteristics of diagnosing urologists were determined from the American Medical Association (AMA) Physician Masterfile. This file maintains a detailed database on the professional and education certifications of > 1.4 million physicians. Characteristics included urologist age, sex, employment status, practice year, and annual radical cystectomy volume. To calculate urologist surgical volume, first we calculated the total number of radical cystectomies performed by each urologist during the study period. This was divided by the number of years urologists performed urologic surgery during the study period, to obtain the annual radical cystectomy volume. We used the 90th percentile cutoff to categorize high versus low volume [15]. At the time of RC, the primary urologist who performed the radical cystectomy was identified as the urologist who was the primary billing urologist at the time of RC.

#### 2.4.3 Hospital characteristics

We assigned each diagnosing urologist to one hospital where he/she performed > 50% of all urologic surgeries [16]. All hospital characteristics were obtained from the SEER-Medicare database. Hospital characteristics included National Cancer Institute affiliation, teaching hospital type, geographic location (rural vs urban), and type of ownership (nonprofit, government, and proprietary). Hospital volume was calculated as the total number of radical cystectomies performed over the study period divided by the 10 yr, and categorized into low versus high volume based on the 90th percentile cutoff.

### 2.5 Statistical analysis

Descriptive statistics were used to summarize the characteristics of patients, urologists, and hospitals using chi-square tests for categorical variables and *t* tests for continuous variables. We constructed a two-level logistic regression model (patients nested within hospital) to determine the use of radical cystectomy. We did not consider nesting of patients within urologists, as there were a limited number of patients per urologist. The outcome variable in the model was the use of radical cystectomy (yes vs no), and the model included all patient, urologist, and hospital characteristics. Multilevel models were constructed with patient, urologist and hospital characteristics entered as fixed effects and hospital effect entered as a random intercept [17], [18]. Association of patient, urologist, and hospital factors with the use of radical cystectomy was reported using odds ratios (ORs) and 95% confidence intervals (CIs).

To determine variation across hospitals in radical cystectomy use, we constructed a null two-level model in which we did not include any patient, urologist, or hospital characteristics. Using the latent variable approach, we calculated intraclass correlation coefficient (ICC) as $\frac{\tau^{2}}{\tau^{2}+3.29}$, where $\tau^{2}$ refers to the variance from the random hospital effect; ICC estimates the proportion of variance in radical cystectomy use attributed to hospitals [19]. To determine how much of this variation can be explained, we included patient, urologist, and hospital characteristics and recalculated ICC. From the fully adjusted two-level model, we plotted the mean rate and 95% CI of radical cystectomy use for each hospital to visualize variation across hospitals graphically. All statistical analyses were performed using SAS 9.4 (SAS Institute Inc.).

## 3 Results

The study cohort included 7097 bladder cancer patients (Fig. 1). These patients were diagnosed by 4601 urologists who were affiliated to 822 hospitals. Overall, 1878 (26.5%) patients underwent radical cystectomy. A total of 252 (13.4%) patients received neoadjuvant chemotherapy followed by radical cystectomy. A total of 3234 (45.6%) patients did not undergo radical cystectomy, 1386 (19.5%) received chemotherapy or radiotherapy alone, and 599 (8.4%) underwent trimodal therapy. Among patients who did not undergo radical cystectomy, the median age was 78 (interquartile range [IQR]: 74, 82) yr and CCI was 1 (IQR: 0, 2).

### 3.1 Patient characteristics

A significantly higher percentage of patients who underwent radical cystectomy were younger, female, and married, and had minimal comorbidities (CCI score < 1) compared with those who did not undergo radical cystectomy (all *p* < 0.01; Table 1).

**Table 1:** Patient characteristics.

	All patients	Radical cystectomy	No radical cystectomy	*p v*alue
	*N*	*N* (%)	*N* (%)	
Age group (yr)				
66–69	854	391 (20.8)	463 (8.9)	< 0.001
70–74	1269	511 (27.2)	758 (14.5)	
75–79	1595	515 (27.4)	1080 (20.7)	
80–85	3379	461 (24.6)	2918 (55.9)	
Sex				
Male	4685	1177 (62.7)	3508 (67.2)	0.004
Female	2412	701 (37.3)	1711 (32.8)	
Race				
Non-Hispanic white	6197	1645 (87.6)	4552 (87.2)	< 0.001
Non-Hispanic black	391	72 (3.8)	319 (6.1)	
Hispanic	195	69 (3.7)	126 (2.4)	
Other	314	92 (4.9)	222 (4.3)	
Marital status				
Married	3828	1186 (63.2)	2642 (50.6)	< 0.001
Single	949	249 (13.3)	700 (13.4)	
Unknown	2320	443 (23.6)	1877 (36.0)	
Census region				
West	2708	766 (40.8)	1942 (37.2)	0.047
Northeast	1808	460 (24.5)	1348 (25.8)	
Midwest	832	217 (11.6)	615 (11.8)	
South	1749	435 (23.2)	1314 (25.2)	
Tumor stage				
II	3984	740 (39.4)	3244 (62.2)	< 0.001
III	1297	584 (31.1)	713 (13.7)	
IV	1816	554 (29.5)	1262 (24.2)	
Tumor grade				
Low	456	80 (4.3)	376 (7.2)	< 0.001
High	6284	1750 (93.2)	4534 (86.9)	
Unknown	357	48 (2.6)	309 (5.9)	
Comorbidity score				
0	3353	1073 (57.1)	2280 (43.7)	< 0.001
1	1833	488 (26.0)	1345 (25.8)	
2	921	185 (9.9)	736 (14.1)	
3+	990	132 (7.0)	858 (16.4)	
Education level (%) a				
≤20.58	1820	525 (28.0)	1295 (24.8)	0.002
20.59–27.36	1672	467 (24.9)	1205 (23.1)	
27.37–34.83	1789	427 (22.7)	1362 (26.1)	
≥34.84	1816	459 (24.4)	1357 (26.0)	
Year of diagnosis				
2002	720	203 (10.8)	517 (9.9)	0.156
2003	685	204 (10.9)	481 (9.2)	
2004	772	210 (11.2)	562 (10.8)	
2005	807	225 (12.0)	582 (11.2)	
2006	728	189 (10.1)	539 (10.3)	
2007	697	175 (9.3)	522 (10.0)	
2008	707	191 (10.2)	516 (9.9)	
2009	651	154 (8.2)	497 (9.5)	
2010	688	178 (9.5)	510 (9.8)	
2011	642	149 (7.9)	493 (9.5)	

a Educational level: the percentage of residents who had at least 4 yr of college education.

### 3.2 Diagnosing urologist characteristics

Diagnosing urologists with a surgeon volume of 0–4 versus 5+, 1666 (25.4%) versus 212 (37.9%) patients underwent radical cystectomy. Diagnosing urologists who performed radical cystectomies were younger (*p* = 0.002), in practice for a shorter period of time (*p* = 0.001), and associated with hospitals that had a volume of fewer than five cases (88.7%; *p* < 0.001) over the study period (Table 2).

**Table 2:** Diagnosing urologist and hospital characteristics.

	Diagnosing urologist			
	Total patients	Radical cystectomy	No radical cystectomy	*p v*alue
		*N* (%)	*N* (%)	
*Urologist characteristics*				
Age of physician, mean (SD)	NA	51.7 (10.0)	52.6 (10.0)	0.002
Practice year, mean (SD)	NA	24.8 (10.5)	25.7 (10.4)	0.001
Physician sex				
Male	6438	1662 (88.5)	4776 (91.5)	0.001
Female	659	216 (11.5)	443 (8.5)	
Employment				
Group	3858	1051 (56.0)	2807 (53.8)	0.304
1–2 physicians	2078	515 (27.4)	1563 (30.0)	
Government	505	127 (6.8)	378 (7.2)	
Medical school	43	13 (0.7)	30 (0.6)	
Nongovernment	129	34 (1.8)	95 (1.8)	
Not classified	484	138 (7.4)	346 (6.6)	
Surgeon volume a				
Low (0–4)	6538	1666 (88.7)	4872 (93.4)	< 0.001
High (5+)	559	212 (11.3)	347 (6.6)	
*Hospital characteristics*				
Bed size, mean (SD)	NA	373.9 (250.3)	347.5 (235.2)	< 0.001
Cancer center b				
No	6615	1689 (90.0)	4926 (94.4)	< 0.001
Clinical	141	44 (2.3)	97 (1.9)	
Comprehensive	341	145 (7.7)	196 (3.7)	
Teaching hospital				
Yes	3798	1060 (56.4)	2738 (52.5)	0.003
No	3299	818 (43.6)	2481 (47.5)	
Type of control				
Nonprofit	5322	1391 (74.1)	3931 (75.3)	0.533
Government	871	236 (12.6)	635 (12.2)	
Proprietary	904	251 (13.4)	653 (12.5)	
Rural/urban				
Rural	818	186 (9.9)	632 (12.1)	0.010
Urban	6279	1692 (90.1)	4587 (87.9)	
Hospital volume a				
Low (0–4)	5786	1406 (74.9)	4380 (83.9)	< 0.001
High (5+)	1311	472 (25.1)	839 (16.1)	

NA = not available; SD = standard deviation.

a Surgeon volume and hospital volume were for the study period (10 yr).

b National Cancer Institute cancer center affiliation.

### 3.3 Hospital characteristics

Radical cystectomy was performed in hospitals with greater bed capacity and cancer center affiliation (both *p* < 0.001). A greater proportion of radical cystectomies were performed at teaching versus nonteaching hospitals (56.4% vs 43.6%). A greater proportion of hospitals that performed radical cystectomy were of high volume (more than five cases) versus those performing no radical cystectomy (25.1% vs 16.1%, *p* < 0.001).

### 3.4 Association of patient, diagnosing urologist, and hospital characteristics with the use of radical cystectomy

Table 3 reports adjusted ORs from the two-level logistic regression model. Patients who were older and had greater comorbidities were less likely to undergo radical cystectomy. Greater diagnosing urologists’ radical cystectomy volume (more than five vs zero to four surgeries; OR, 1.27; 95% CI, 1.00–1.62) and hospital volume (more than five vs zero to four surgeries; OR, 1.48; 95% CI, 1.14–1.93) were associated with higher radical cystectomy use. Radical cystectomy was more likely performed when a patient was seen by a female than when the patient was seen by a male urologist (OR, 1.32; 95% CI, 1.07–1.62). Additionally, we performed an interaction analysis and found female patients were more likely to receive a radical cystectomy when seen by a female urologist (OR, 1.80; 95% CI, 1.37–2.38). There was no significant interaction between female urologists and male patients (OR, 0.92; 95% CI, 0.68–1.25).

**Table 3:** Association of patient, diagnosing urologist, and hospital characteristics with radical cystectomy use.

Characteristics	Odds ratio (95% CI)
*Patient*	
Age group (yr)	
66–69	Ref
70–74	0.81 (0.67–0.99)
75–79	0.57 (0.47–0.69)
80–85	0.19 (0.15–0.23)
Sex	
Male	Ref
Female	1.63 (1.42–1.87)
Race	
Non-Hispanic white	Ref
Non-Hispanic black	0.59 (0.44–0.79)
Hispanic	1.33 (0.93–1.90)
Other	0.96 (0.72–1.28)
Marital status	
Married	Ref
Single	0.61 (0.54–0.73)
Unknown	0.63 (0.54–0.73)
Census region	
West	Ref
Northeast	0.93 (0.75–1.14)
Midwest	0.87 (0.67–1.14)
South	0.89 (0.72–1.10)
Tumor stage	
II	Ref
III	3.61 (3.10–4.21)
IV	1.59 (1.38–1.83)
Tumor grade	
Low	Ref
High	1.92 (1.46–2.54)
Unknown	0.81 (0.53–1.23)
Comorbidity score	
0	Ref
1	0.82 (0.71–0.94)
2	0.60 (0.49–0.73)
3+	0.36 (0.29–0.45)
Education level (%) a	
≤20.58	Ref
20.59–27.36	1.03 (0.87–1.23)
27.37–34.83	0.84 (0.69–1.01)
≥34.84	0.98 (0.79–1.20)
Year of diagnosis	
2002	1.04 (0.79–1.38)
2003	1.13 (0.85–1.49)
2004	1.21 (0.92–1.60)
2005	1.25 (0.95–1.64)
2006	1.14 (0.86–1.51)
2007	1.10 (0.82–1.46)
2008	1.15 (0.87–1.53)
2009	1.01 (0.75–1.35)
2010	1.14 (0.85–1.51)
2011	Ref
*Diagnosing urologist*	
Age of physician	0.99 (0.97–1.02)
Practice year	1.00 (0.98–1.03)
Physician sex	
Male	Ref
Female	1.32 (1.07–1.62)
Employment	
Group	Ref
1–2 physicians	0.97 (0.83–1.12)
Government	0.68 (0.53–0.87)
Medical school	0.78 (0.36–1.67)
Nongovernment	0.91 (0.57–1.45)
Not classified	0.95 (0.74–1.23)
Surgeon volume b	
Low (0–4)	Ref
High (5+)	1.27 (1.00–1.62)
*Hospital*	
Bed size	1.00 (1.00–1.00)
Cancer center c	
No	Ref
Clinical	0.78 (0.46–1.33)
Comprehensive	1.32 (0.89–1.96)
Teaching hospital	
No	Ref
Yes	1.01 (0.85–1.20)
Type of control	
Nonprofit	Ref
Government	1.07 (0.86–1.34)
Proprietary	1.12 (0.90–1.38)
Rural/urban	
Urban	Ref
Rural	0.89 (0.70–1.14)
Hospital volume b	
Low (0–4)	Ref
High (5+)	1.48 (1.14–1.93)

CI = confidence interval; Ref = reference.

a Educational level: the percentage of residents who had at least 4 yr of college education.

b Surgeon volume and hospital volume were for the study period (10 yr).

c National Cancer Institute (NCI) cancer center affiliation.

### 3.5 Variation in the use of radial cystectomy attributed to hospitals

**Fig. 2:**
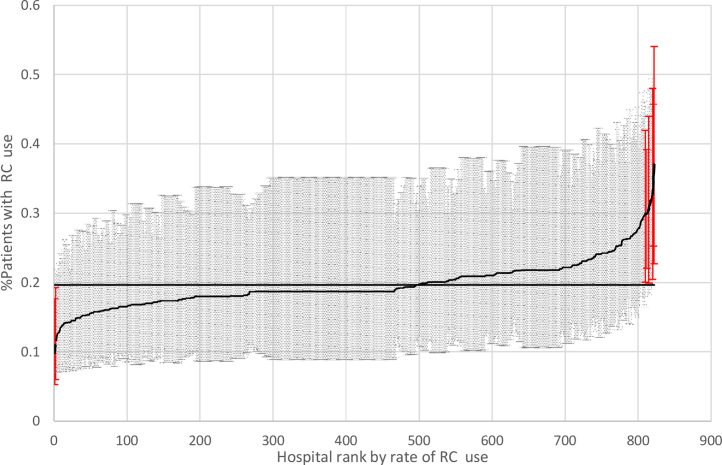
Hospital-specific rates of radical cystectomy use by hospital rank. Rates of radical cystectomy use were based on the two-level hierarchical model. Rates of radical cystectomy use for 822 hospitals were ranked from lowest to highest. The rates were calculated using hierarchical generalized linear models (two levels), adjusted for patient, diagnosing urologist, and hospital characteristics. The horizontal line represents the overall mean rate of radical cystectomy use. Error bars represent 95% confidence intervals for the rates of individual hospital. RC = radical cystectomy.

In the null two-level logistic regression model, the ICC for variation in the use of radical cystectomy attributed to the hospital level was 6.4%. After controlling for all patient, urologist, and hospital characteristics, the ICC reduced to 4.3%. Fig. 2 depicts the hospital-level–adjusted radical cystectomy utilization rate ranked from lowest to highest along with 95% CIs. Hospital-level radical cystectomy use varied from 9.9% to 37.0%, with a mean rate of 19.7%. Out of 822 hospitals, only two had lower use of radical cystectomy below the mean and one had higher use above the mean (*p* < 0.05).

## 4 Discussion

Despite being a long-standing guideline-recommended treatment, radical cystectomy remains vastly underutilized in the USA. In the present study, increased hospital volume was associated with increased use of radical cystectomy; however, variation in the use of radical cystectomy at the hospital level was only 4.3%. We identified that increased surgical volume of diagnosing urologists was associated with increased use of radical cystectomy. Furthermore, female urologists were more likely to perform radical cystectomy.

Our study has several important findings. First, we found that hospital volume impacted the use of radical cystectomy. Moreover, variation in radical cystectomy use attributed to the hospital level was small. Hospital volume has frequently been used as a quality metric for complex surgical procedures [20]. A recent paper by Bruins et al [21] showed that hospitals should perform > 20 radical cystectomies per year, according to which our study would include only two out of the 822 hospitals. However, this and prior radical cystectomy studies determining the relationship between volume outcomes and hospitals have been derived from tertiary care referral centers, cancer centers, and/or centers associated with treating urologists (ie, radical cystectomists) and not diagnosing urologists [22]. Furthermore, in our study, the rationale for five radical cystectomies per year was based on prior SEER studies, in which the 90% threshold value was 5/yr [23]. In the present study, diagnosing urologists’ primary practice was largely located in nonacademic centers that generally had lower volumes of radical cystectomy. A possible explanation for hospital volume impacting diagnosing urologists was that higher-volume hospitals were more likely to harbor higher-volume urologists in combination with hospitals well equipped with advanced care processes. Higher-volume hospitals often manage complex surgeries and potential complications (ie, access to interventional radiology, specialty consultants, and surgical intensive care units). This access to a breadth of ancillary services at higher-volume hospitals has previously been established as a mechanism for improved surgical outcomes [24]. Additional patient characteristics associated with lower radical cystectomy utilization include older age (>70 yr), male sex, and non-Hispanic black ethnicity with multiple comorbidities. These are not novel findings, but confirmed findings seen in prior studies [4]. Future efforts on increasing radical cystectomy utilization may target improving the surrounding infrastructure at lower-volume hospitals that harbor higher-volume urologists.

Second, we found that patients treated with high-volume diagnosing urologists were more likely to have undergone a radical cystectomy. This is the first study to our knowledge that assessed the impact of the diagnosing urologist on the use of radical cystectomy. As confirmed in other malignancies, specialists have previously been shown to recommend the therapy that they themselves deliver [25]. As previous papers have shown, there has been marginal improvement in the underutilization of radical cystectomy in the past 2 decades [3], [4]. We found that only 26.5% patients with muscle-invasive bladder cancer underwent radical cystectomy. While we cannot quantitate the variation attributed by the diagnosing urologist, > 95% variation in the use of radical cystectomy was attributed to other “nonhospital” factors. This present study suggests that the diagnosing urologist and in particular his/her surgical volume should be considered in the framework for the centralization of these complex surgeries in an attempt to increase the utilization of radical cystectomy as observed in other countries.

Third, we found that female urologists were more likely to perform radical cystectomy than male urologists. Other studies have found provider-specific sex difference in mortality rates, with patients treated by female surgeons to have significantly decreased mortality rates as compared with those treated by male surgeons [26]. Herein, we found increased use of a high-risk surgery, which carries non-negligible morbidity and mortality, among female urologists. These findings come on the heels of a changing demographic landscape in which female urologists have grown from < 2% in 1995 to nearly 9% of the workforce in more recent years. Our study showed that concordance between urologist sex and sex of the patient increased radical cystectomy utilization between female patients seen by female urologists. This finding serves as a potential opportunity to improve the utilization of radical cystectomy in line with prior studies that have shown that gender concordance between providers and patients can improve clinical outcomes [27]. Despite a significant proportion of women receiving a radical cystectomy, there was a dearth of female urologists, which might in part explain the overall underutilization of radical cystectomy. Perhaps the consideration of gender concordance between patients and provider might be another method to improve the utilization of radical cystectomy. Furthermore, other studies have tried to elucidate the characteristics of female physicians, which confer an advantage with a myriad of conclusions that might shed light on our findings. There is some evidence that female physicians are more likely to have a participatory emphasis during the clinic visit utilizing clear and positive communication, while also more consistently providing supplemental resources compared with their male colleagues [28]. In the present study, female urologists were younger than their male counterparts. Prior studies have suggested that as surgeons age, they are more likely to reduce case complexity and patient volume, which would translate into decreased utilization of radical cystectomy [29]. Presumed advantages of this younger female cohort includes increased subspecialty training, comfort with advanced surgical approaches (open, laparoscopic, and robotic), and increased familiarity with updated bladder cancer guidelines [30]. Future studies are warranted to explain other characteristics that may drive differences in radical cystectomy utilization according to urologist sex.

Finally, it is important to emphasize that, given the complexity in managing these patients, a multidisciplinary approach should be taken. While the SEER-Medicare database lacks the granularity to assess the role of a multidisciplinary team in the utilization of radical cystectomy, we recognize the importance of a multidisciplinary team consisting of at least a urologic oncologist, a medical oncologist, and a radiation oncologist from a referral center providing input on the indications for a radical cystectomy.

Our findings must be interpreted within the context of our study design. First, this is a retrospective cohort study with inherent selection bias. However, we provide a generalizable nationwide cohort to describe variation in practice patterns using hierarchical models. Ignoring a level of nesting data can impact estimated variances and the available power to detect treatment or covariate effect, which can seriously inflate type I error rates, leading to substantive errors when interpreting results. While multilevel modeling accounts for nesting of data, we observed limited numbers of patients/hospital to be relatively small, which reduced the variability of rate across hospitals due to the shrinkage estimation. Second, the use of Medicare claims data was limited to patients aged 65 yr and older. Therefore, our findings may not be applicable to younger patients; however, bladder cancer is more commonly diagnosed in the elderly with a median age at diagnosis of 72 yr. Third, we cannot determine critical components in the treatment decision-making process. We were unable to differentiate between patient refusal to consider surgery, willingness to visit a high-volume urologist, lack of referral, loss to follow-up, or whether their care was paid for by another insurer. Fourth, we were unable to determine variation at the urologist level in our multilevel model due to limited numbers of patients per urologist. However, the relatively robust dataset provided by the SEER-Medicare linked with the AMA Physician Masterfile allowed us to identify a large number of urologists and individual characteristics from a relatively large nationwide sample, to describe practice patterns in the use of radical cystectomy. Moreover, the present study allowed us to assess variation attributed to hospital characteristics and suggests that further investigation into other “nonhospital” factors is needed to improve the use of radical cystectomy. Fifth, we acknowledge that while difference in the age of urologists was significantly associated with radical cystectomy use, clinical relevance of urologist age is not clearly established. Finally, in calculating the number of cystectomies per urologist, we used a prior established methodology when assigning patients who were seen by two or more urologists [14]. Furthermore, at the time of RC, the primary urologist who performed the radical cystectomy was identified as the urologist who was the primary billing urologist at the time of RC. A limitation of this methodology includes loss of generalizability, for example, in group practices where cystectomies are typically performed by two urologists.

## 5 Conclusions

We found that 4.3% variation in the use of radical cystectomy was attributed to hospitals, with higher hospital volume being the main driver for increased utilization. We also identified significantly increased use among higher-volume and female diagnosing urologists. These findings support further investigation into measures beyond hospital volume, which largely impact the utilization of radical cystectomy.

## 6 Credit Author Statement

**Vishnukamal** **Golla** (Conceptualization; Funding acquisition; Investigation; Methodology; Visualization; Roles/Writing – original draft; Writing – review & editing), **Yong Shan** (Conceptualization; Data curation; Formal analysis; Investigation; Methodology; Project administration; Resources; Software; Validation; Visualization; Roles/Writing – original draft; Writing – review & editing), **Hemalkumar B. Mehta** (Conceptualization; Data curation; Formal analysis; Funding acquisition; Investigation; Methodology; Project administration; Resources; Software; Supervision; Validation; Visualization; Roles/Writing – original draft; Writing – review & editing), **Zachary Klaassen** (Roles/Writing – original draft; Writing – review & editing), **Douglas S. Tyler** (Project administration; Resources; Software; Supervision; Validation; Visualization; Roles/Writing – original draft; Writing – review & editing), **Jacques Baillargeon** (Conceptualization; Investigation; Methodology; Supervision; Validation; Visualization; Roles/Writing – original draft; Writing – review & editing), **Ashish M. Kamat** (Conceptualization; Investigation; Methodology; Visualization; Roles/Writing – original draft; Writing – review & editing), **Stephen J. Freedland** (Roles/Writing – original draft; Writing – review & editing), **John L. Gore** (Conceptualization; Methodology; Visualization; Roles/Writing – original draft; Writing – review & editing), **Karim Chamie** (Conceptualization; Investigation; Methodology; Project administration; Supervision; Validation; Visualization; Roles/Writing – original draft; Writing – review & editing), **Yong-Fang Kuo** (Conceptualization; Data curation; Formal analysis; Investigation; Methodology; Project administration; Resources; Software; Supervision; Validation; Visualization; Roles/Writing – original draft; Writing – review & editing), **Stephen B. Williams** (Conceptualization; Data curation; Formal analysis; Funding acquisition; Investigation; Methodology; Project administration; Resources; Software; Supervision; Validation; Visualization; Roles/Writing – original draft; Writing – review & editing)

***Author contributions*****:** Stephen B. Williams had full access to all the data in the study and takes responsibility for the integrity of the data and the accuracy of the data analysis.

*Study concept and design*: Golla, Shan, Mehta, Chamie, Kuo, Williams.

*Acquisition of data*: Shan, Mehta, Kuo, Williams.

*Analysis and interpretation of data*: Golla, Shan, Mehta, Klaassen, Tyler, Baillargeon, Kamat, Freedland, Gore, Chamie, Kuo, Williams.

*Drafting of the manuscript*: Golla, Shan, Mehta, Klaassen, Tyler, Baillargeon, Kamat, Freedland, Gore, Chamie, Kuo, Williams.

*Critical revision of the manuscript for important intellectual content*: Golla, Shan, Mehta, Klaassen, Tyler, Baillargeon, Kamat, Freedland, Gore, Chamie, Kuo, Williams.

*Statistical analysis*: Shan, Mehta, Kuo.

*Obtaining funding*: Williams.

*Administrative, technical, or material support*: Williams.

*Supervision*: Mehta, Chamie, Kuo, Williams.

*Other*: None.

***Financial disclosures:*** Stephen B. Williams certifies that all conflicts of interest, including specific financial interests and relationships and affiliations relevant to the subject matter or materials discussed in the manuscript (eg, employment/affiliation, grants or funding, consultancies, honoraria, stock ownership or options, expert testimony, royalties, or patents filed, received, or pending), are the following: None.

***Funding/Support and role of the sponsor*****:** This study was funded by the Kahlert Foundation and the American Urological Association (AUA) Residency Research Award. This study was conducted with the support of a Department of Defense Peer Reviewed Cancer Research Program (PRCRP) Career Development Award (W81XWH1710576) (SBW). The funding sources were used for the design and conduct of the study. The funding sources had no role in the collection, management, analysis, and interpretation of the data; preparation, review, or approval of the manuscript; and decision to submit the manuscript for publication. The content is solely the responsibility of the authors and does not necessarily represent the official views of the National Institutes of Health.

***Acknowledgments*****:** This study used the Surveillance, Epidemiology, and End Results (SEER)-Medicare linked database. The interpretation and reporting of these data are the sole responsibility of the authors. The authors acknowledge the efforts of the Applied Research Program, NCI; the Office of Research, Development and Information, CMS; Information Management Services (IMS), Inc.; and the (SEER) program tumor registries in the creation of the SEER database.
